# Tyrosine kinase fusion genes in pediatric *BCR-ABL1*-like acute lymphoblastic leukemia

**DOI:** 10.18632/oncotarget.13492

**Published:** 2016-11-22

**Authors:** Judith M. Boer, Elisabeth M.P. Steeghs, João R.M. Marchante, Aurélie Boeree, James J. Beaudoin, H. Berna Beverloo, Roland P. Kuiper, Gabriele Escherich, Vincent H.J. van der Velden, C. Ellen van der Schoot, Hester A. de Groot-Kruseman, Rob Pieters, Monique L. den Boer

**Affiliations:** ^1^ Department of Pediatric Oncology/Hematology, Erasmus Medical Center, Sophia Children's Hospital, Rotterdam, The Netherlands; ^2^ Department of Clinical Genetics, Erasmus Medical Center, Rotterdam, The Netherlands; ^3^ Dutch Childhood Oncology Group (DCOG), The Hague, The Netherlands; ^4^ Department of Human Genetics, Radboud University Medical Center and Radboud Institute for Molecular Life Sciences, Nijmegen, The Netherlands; ^5^ German Cooperative Study Group for Childhood Acute Lymphoblastic Leukemia (COALL), Hamburg, Germany; ^6^ Department of Immunology, Erasmus Medical Center, Rotterdam, The Netherlands; ^7^ Sanquin Research and Landsteiner Laboratory, Academic Medical Centre, University of Amsterdam, Amsterdam, The Netherlands; ^8^ Princess Máxima Center for Pediatric Oncology, Utrecht, The Netherlands

**Keywords:** BCR-ABL1-like, pediatric B cell precursor acute lymphoblastic leukemia, tyrosine kinase fusion, minimal residual disease

## Abstract

Approximately 15% of pediatric B cell precursor acute lymphoblastic leukemia (BCP-ALL) is characterized by gene expression similar to that of *BCR-ABL1*-positive disease and unfavorable prognosis. This *BCR-ABL1*-like subtype shows a high frequency of B-cell development gene aberrations and tyrosine kinase-activating lesions. To evaluate the clinical significance of tyrosine kinase gene fusions in children with BCP-ALL, we studied the frequency of recently identified tyrosine kinase fusions, associated genetic features, and prognosis in a representative Dutch/German cohort. We identified 14 tyrosine kinase fusions among 77 *BCR-ABL1*-like cases (18%) and none among 76 non-*BCR-ABL1*-like B-other cases. Novel exon fusions were identified for *RCSD1-ABL2* and *TERF2-JAK2*. *JAK2* mutation was mutually exclusive with tyrosine kinase fusions and only occurred in cases with high *CRLF2* expression. The non/late response rate and levels of minimal residual disease in the fusion-positive *BCR-ABL1*-like group were higher than in the non-*BCR-ABL1*-like B-others (p<0.01), and also higher, albeit not statistically significant, compared with the fusion-negative *BCR-ABL1*-like group. The 8-year cumulative incidence of relapse in the fusion-positive *BCR-ABL1*-like group (35%) was comparable with that in the fusion-negative *BCR-ABL1*-like group (35%), and worse than in the non-*BCR-ABL1*-like B-other group (17%, p=0.07). *IKZF1* deletions, predominantly other than the dominant-negative isoform and full deletion, co-occurred with tyrosine kinase fusions. This study shows that tyrosine kinase fusion-positive cases are a high-risk subtype of BCP-ALL, which warrants further studies with specific kinase inhibitors to improve outcome.

## INTRODUCTION

Children with B-cell precursor acute lymphoblastic leukemia (BCP-ALL) with the *BCR-ABL1* fusion gene form a small patient group with a poor prognosis, which has been substantially improved in recent treatment protocols with the addition of imatinib and other tyrosine kinase inhibitors [1–2]. Approximately 15% of cases of BCP-ALL are characterized by a gene expression signature similar to that of *BCR-ABL1*-positive disease and an unfavorable prognosis [3–4]. This *BCR-ABL1*-like subtype is found in approximately 50% of so-called B-other cases, which are BCP-ALL cases negative for the sentinel cytogenetic lesions *BCR-ABL1*, *ETV6-RUNX1*, *TCF3-PBX1*, rearrangement of *MLL*, or high hyperdiploidy (51-65 chromosomes). *BCR-ABL1*-like BCP-ALL shows a high frequency of B-cell development gene aberrations, especially *IKZF1* deletions [3–5], and tyrosine kinase activating lesions [6–7].

Tyrosine kinase activating lesions are diverse and include tyrosine kinase fusion proteins, cytokine receptor overexpression, or activating point mutations in genes encoding kinases, cytokine receptors, or signaling molecules (recently reviewed in [8]). In our study, we focus on tyrosine kinase fusion genes because they are most similar to the well-known *BCR-ABL1* fusion gene and expected to be clonal leukemia drivers. Detection of tyrosine kinase fusions could guide targeted treatment with tyrosine kinase inhibitors and improve outcome in a similar way as currently for *BCR-ABL1*-positive patients.

Tyrosine kinases known to be involved in fusions include the ABL class kinases ABL1, ABL2, PDGFRB and CSF1R as well as the JAK class kinase JAK2. Each of these kinases has been detected in in-frame fusions with multiple 5′ partner genes resulting in the expression of a constitutively active, oncogenic kinase. The number of patients without sentinel chromosomal abnormalities and the diversity of novel tyrosine kinase fusions poses a challenge to routine fusion gene detection. Previous studies have described the discovery and oncogenic potential of ABL and JAK class fusion genes and the sensitivity of patients’ cells or *in vitro* cell models to tyrosine kinase inhibition [6–7]. Selection of patients for fusion detection in these studies was based on gene expression profiling indicating Philadelphia-like ALL [6–7]. A large Japanese study screened all B-other cases by transcriptome analysis or multiplexed RT-PCR for 15 fusions and found both methods similarly sensitive [9]. Other studies describe systematic screens in B-other cases by FISH or RT-PCR to detect specific tyrosine kinase fusions [10–11].

We aimed at the detection of recently identified tyrosine kinase fusion genes in 153 B-other cases of a population-based cohort of 574 Dutch/German pediatric BCP-ALL patients at initial diagnosis to address the frequency of tyrosine kinase fusions, their clinical response characteristics, and associated genetic lesions in B-cell development genes.

## RESULTS

### Tyrosine kinase fusions are restricted to *BCR-ABL1*-like subtype

We identified 14 ABL/JAK class tyrosine kinase activating fusion genes among 77 *BCR-ABL1*-like cases (18%), and none among 76 non-*BCR-ABL1*-like B-other cases (Table 1; Supplementary Table 1; Supplementary Figure 1). We found nine tyrosine kinase fusions predictive for activated ABL signaling: 4 *EBF1-PDGFRB*, 2 *SSBP1-CSF1R*, and one each of *ZMIZ1-ABL1*, *FOXP1-ABL1*, *RCSD1-ABL2*. Five tyrosine kinase fusions are predictive for activated JAK signaling: 3 *PAX5-JAK2*, one each of *BCR-JAK2* and *TERF2-JAK2*. The exons included in the fusion transcripts were the same as described previously [6, 12–13], except for *RCSD1* exon 3-*ABL2* exon 5 (Supplementary Figure 2), and *TERF2* exon 10-*JAK2* exon 19 with a deletion of the last 11 coding nucleotides of *TERF2* exon 10 (Supplementary Figure 3). All detected fusion transcripts encode in-frame fusion proteins as evaluated using ProteinPaint [14].

**Table 1:** Frequency of identified tyrosine kinase fusion genes

Marker	*BCR-ABL1*-like (n=77)	Remaining B-other (n=76)
***ABL1/ABL2* fusion**	**3.9%**	0%
*ZMIZ1-ABL1*	1	
*FOXP1-ABL1*	1	
*RCSD1-ABL2*	1	
***PDGFRB* fusion**	**5.2%**	0%
*EBF1-PDGFRB*	4	
***CSF1R* fusion**	**2.6%**	0%
*SSBP2-CSF1R*	2	
***JAK2* fusion**	**6.5%**	0%
*PAX5-JAK2*	3	
*BCR-JAK2*	1	
*TERF2-JAK2*	1	
*CRLF2* high expression*	15.6%	15.8%
PAR1 deletion**	10.5%	10.7%

* Expression of Affymetrix U133 Plus 2.0 probe set 208303_s_at above the 90th percentile of the total BCP-ALL group, as described previously [5].

** Deletion of *IL3RA* and *CSF2RA* and retention of *CRLF2* as determined by MLPA.

Table 2 summarizes the genomic analyses of the tyrosine kinase fusion cases. All *PDGFRB*, *CSF1R* and *ABL1* fusions showed aberrant FISH patterns with the appropriate break apart FISH probes (Table 2, Supplementary Figure 4). In addition, both *ABL1* fusions showed add(9)(q34) in their karyotypes (*ABL1* is located on 9q34), together with add(10)(q21) in the *ZMIZ1-ABL1* case (*ZMIZ1* is located on 10q22), and with del(3)(p13) in the *FOXP1-ABL1* case (*FOXP1* is located on 3p13). Two *EBF1-PDGFRB* cases showed an interstitial 5q deletion on array-CGH, one case arose by balanced t(5;5) translocation, and one case showed a more complex copy number alteration, with loss of *EBF1* exon 16 and gain of a genomic region encompassing *EBF1* exons 7-9 (Table 2; Supplementary Figure 5A-B). *EBF1* exon 16 deletion was confirmed by MLPA in the two interstitial 5q deletion cases and the complex case (Table 2). Genomic breaks were also detected in *SSBP2-CSF1R*, *BCR2-JAK2* and *TERF2-JAK2* fusions (Table 2, Supplementary Figure 5C-F). Interestingly, one *SSBP2-CSF1R* fusion probably arose by chromothripsis of chromosome 5 (Supplementary Figure 5D). Finally, when we ordered the gene expression levels of the involved tyrosine kinases for the 153 B-other BCP-ALL cases, the expression in the fusion cases ranked in the top (median percentile 2.6%, range 0.7-14%; Table 2).

**Table 2:** Molecular characteristics of the identified tyrosine kinase fusion genes

Case	Tyrosine kinase fusion	Simplified karyotype	Array-CGH^a^	*EBF1* exon 16^b^	FISH^c^	Expr rank^d^	Exons fused	Validation
R32	*EBF1-PDGFRB*	ND	deletion chr5: 149,494,702-158,058,047	0.64	*PDGFRB* split	1%	e15-e11	RT-PCR
A288	*EBF1-PDGFRB*	46,XY,t(2;12)(q23~24; q12)[20]/46,XY[14]	diploid	ND	*PDGFRB* split	5%	e14-e11	RT-PCR
A472	*EBF1-PDGFRB*	46,XY[20]	deletion chr5: 149,494,702-158,030,723; gain chr5: 158,049,831-158,428,865	0.62	*PDGFRB* split	4%	e15-e11	RT-PCR
A428	*EBF1-PDGFRB*	46,XY[20].ish 7(cep7,D7S522)x1,i(7)(q10)(D7S522+, cep7+, D7S522+)[13/25]	gain chr5: 158,222,469-158,428,865	0.54	*PDGFRB* split	6%	e15-e11	RT-PCR
A123	*SSBP2-CSF1R*	ND	small deletions typical of chromothripsis	1.0	*PDGFRB* split	1%	e16-e12	RT-PCR
A526	*SSBP2-CSF1R*	46,XY[10]	gain chr5: 80,740,416-149,403,322	1.1	*PDGFRB* split	3%	e16-e12	RT-PCR
A91	*ZMIZ1-ABL1*	47,XY,add(9)(q34), add(10)(q21),mar[9]	diploid	1.1	*ABL1* split	2%	e18-e2	RT-PCR
A26	*FOXP1-ABL1*	46,XY,del(3)(p13), add(9)(q34),i(22)(q10), inc[6]	diploid	1.0	*ABL1* split	14%	e27-e4	TLA; PCR
A530	*RCSD1-ABL2*	46,XY[4]/42~ 46,XY,inc[3]	ND	0.71	ND	1%	e3-e5	RT-PCR; TLA
A31	*PAX5-JAK2*	46,XX,del(1)(q3?2q4?4), inc[2]/46,XX[18]	diploid	0.94	ND	1%	e5-e19	RT-PCR
A286	*PAX5-JAK2*	46,XX[17]	diploid	0.81	ND	6%	e5-e19	RT-PCR
A204	*PAX5-JAK2*	ND	diploid	0.96	ND	3%	e5-e19	RT-PCR
A214	*TERF2-JAK2*	46,XY[20]	gain chr16: 67,958,182-88,690,630;deletion chr9: 593,494-5,068,342	0.95	ND	3%	e10-e19	RT-PCR; TLA
A216	*BCR-JAK2*	ND	deletion chr9: 194,193-5,068,342; gain chr9: 5,080,443-5,647,733;gain chr22: 14,513,474-21,885,107; deletion chr22: 21,898,315-22,667,667	0.91	ND	1%	e1-e17	RT-PCR; TLA

^a^Genome positions in build hg18, copy number changes shown for the genes involved in the fusion; see also Supplementary Figure 5.

^b^*EBF1* exon 16 copy number ratio determined by MLPA. A value below 0.75 is called as a deletion.

^c^FISH break apart probes from Cytocell were used. The centromeric *PDGFRB* probe overlaps with *CSF1R* and can be used to detect breakpoints in *CSF1R* as well as in *PDGFRB*.

^d^Expr rank indicates the percentile in rank of the tyrosine kinase gene expression among 153 B-other BCP-ALL cases on Affymetrix U133 Plus 2 arrays.

ND, not determined.

### *CRLF2* high expression and PAR1 deletions are common in both *BCR-ABL1*-like and B-other cases

**Figure 1:**
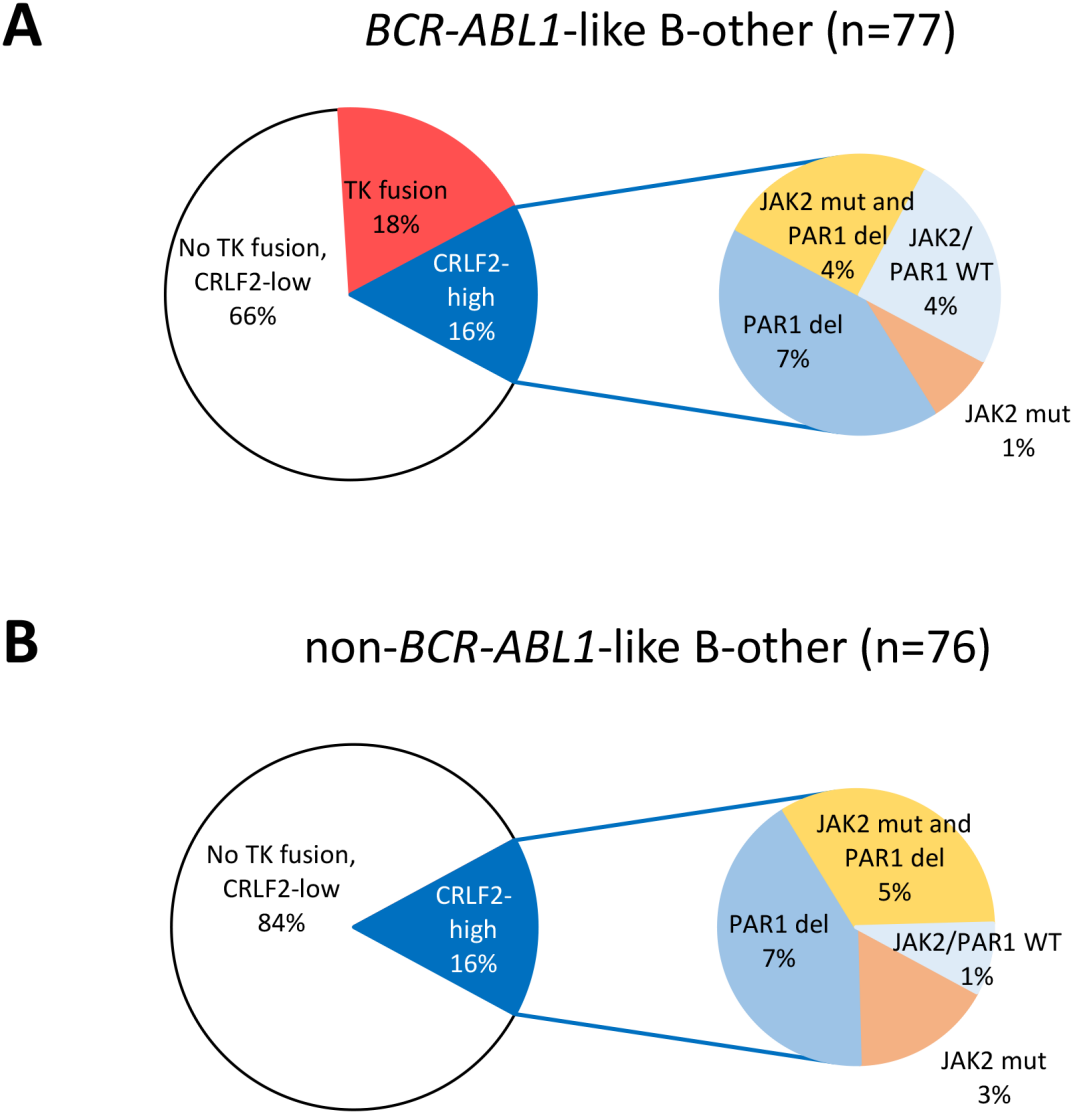
Distribution of tyrosine kinase fusions and *CRLF2* high expression cases Pie diagrams showing the percentages of tyrosine kinase (TK) fusion cases and *CRLF2* high expression among **A**. 77 *BCR-ABL1*-like B-other cases, and **B**. 76 non-*BCR-ABL1*-like B-other cases. Within the *CRLF2* high expression cases, a sub-distribution of cases with *JAK2* mutation and/or PAR1 deletion is shown. In the non-*BCR-ABL1*-like B-other cases, 2 PAR1-deleted cases were not tested for *JAK2* mutations.

High gene expression levels of the cytokine receptor *CRLF2* were found in 16% of *BCR-ABL1*-like cases, none of which overlapped with ABL/JAK class tyrosine kinase fusion cases, and also in 16% of non-*BCR-ABL1*-like B-other cases. About 50% of *CRLF2*-high cases carried a *JAK2* mutation [15]. The frequency of *CRFL2* high expression cases with a PAR1 deletion, *JAK2* mutation or both was similarly high in *BCR-ABL1*-like (9/12 cases) and B-other cases (11/12 cases (Figure 1). *CRLF2* expression had no prognostic value in this Dutch/German BCP-ALL cohort as described previously [5].

### Tyrosine kinase fusion cases are enriched for *IKZF1* deletion variants

Next, we compared the frequency of B-cell development gene lesions between the tyrosine kinase fusion-positive *BCR-ABL1*-like cases, the fusion-negative *BCR-ABL1*-like cases, and the non-*BCR-ABL1*-like B-other cases. While *IKZF1* deletions were common both in fusion-positive and fusion-negative *BCR-ABL1*-like cases (64% vs. 40%; p=0.14), *IKZF1* deletions other than exon 4-7 or full deletion, including deletion of exons 1, 1-2, 1-3, 2-3, 2-7, 2-8, 4-8, 5 and 7-8, occurred more frequently in the fusion-positive compared with the fusion-negative *BCR-ABL1*-like cases (43% vs. 15%; p=0.03; Table 3). Except for deletions in *EBF1*, enriched in *EBF1-PDGFRB* fusion cases due to interstitial 5q deletion, none of the other genes detected by P335 MLPA (*PAX5*, *ETV6*, *RB1*, *BTG1*, *CDKN2A/B*, PAR1) showed an aberrant deletion frequency in tyrosine kinase fusion cases compared with non-*BCR-ABL1*-like B-other cases (Table 2). In the fusion-positive cases, *CDKN2A/B* deletion was less frequent than in the fusion-negative *BCR-ABL1*-like cases (14% vs. 63%, p=0.002; Table 3 ). Two large genomic lesions previously described to occur in *BCR-ABL1*-like BCP-ALL, dic(9;20) and intrachromosomal amplification of chromosome 21 [3, 16], were mutually exclusive with the identified tyrosine kinase fusions (Table 3).

**Table 3:** Clinical and molecular features of ABL/JAK class tyrosine kinase fusions

		TK fusion-positive*BCR-ABL1*-like	TK fusion-negative*BCR-ABL1*-like	Fisher PTK fusion-positive vs. TK fusion-negative *BCR-ABL1*-like	non-*BCR-ABL1*-like B-other	Fisher PTK fusion-positive vs. non-*BCR-ABL1*-like B-other
**Clinical features**						
male		**11/14 (79%)**	**28/63 (44%)**	**0.036**	44/76 (58%)	0.23
age ≥ 10 years		7/14 (50%)	18/63 (29%)	0.2	25/76 (33%)	0.24
WBC ≥ 50×10^9^/L		6/14 (43%)	32/63 (51%)	0.77	23/76 (30%)	0.37
NCI high risk^a^		11/14 (79%)	43/63 (68%)	0.53	42/76 (55%)	0.14
MRD TP1^b^	high intermediate low	7/11 (64%) 3/11 (27%) 1/11 (9%)	17/36 (47%) 4/36 (11%) 15/36 (42%)	0.093	10/45 (22%) 5/45 (11%) 30/45 (67%)	**0.001**
MRD TP2^b^	high intermediate low	4/7 (57%) 0/7 (0%) 3/7 (43%)	4/30 (13%) 3/30 (10%) 23/30 (77%)	**0.037**	1/34 (3%) 1/34 (3%) 32/34 (94%)	**0.002**
Risk arm MR or HR		12/14 (86%)	52/62 (84%)	1	47/74 (64%)	0.13
Prednisone poor response^c^		2/7 (29%)	3/31 (10%)	0.22	3/31 (10%)	0.22
No CR after induction^d^		**5/12 (42%)**	10/58 (17%)	0.11	**5/73 (7%)**	**0.004**
**Molecular features**						
*IKZF1* deletion	Total	**9/14 (64%)**	25/62 (40%)	0.14	**13/73 (18%)**	**0.0009**
	Common^e^	3/14 (21%)	16/62 (26%)	1	5/73 (7%)	0.11
	Other^f^	**6/14 (43%)**	**9/62 (15%)**	**0.026**	**8/73 (11%)**	**0.009**
*EBF1* deletion		**4/14 (29%)**	9/62 (15%)	0.24	**3/73 (4%)**	**0.012**
*EBF1* single exon 16 del		**4/14 (29%)**	**0/62 (0%)**	**0.0008**	**0/73 (0%)**	**0.0005**
*PAX5* deletion/amp		3/14 (21%)	32/62 (52%)	0.072	26/73 (36%)	0.37
*ETV6* deletion		2/14 (14%)	9/62 (15%)	1	18/75 (24%)	0.73
*BTG1* deletion		2/14 (14%)	1/62 (2%)	0.085	6/74 (8%)	0.61
*CDKN2A/B* deletion		**2/14 (14%)**	**39/62 (63%)**	**0.002**	29/75 (39%)	0.13
*RB1* deletion		1/14 (7%)	6/62 (10%)	1	5/74 (7%)	1
*CRLF2* high expression		0/14 (0%)	12/63 (19%)	0.11	12/76 (16%)	0.2
PAR1 deletion		0/14 (0%)	8/62 (13%)	0.34	8/75 (11%)	0.35
dic(9;20)		**0/14 (0%)**	**15/57 (26%)**	**0.031**	2/74 (3%)	1
iAMP21		0/14 (0%)	10/57 (18%)	0.19	2/74 (3%)	1

^a^NCI-Rome high risk is defined by white blood cell count (WBC) ≥_50×10^9^/L and/or age ≥ 10 years.

^b^Minimal residual disease PCR high (≥10^−3^), intermediate (≥10^−4^ and <10^−3^), low (<10^−4^); TP1, after the first induction course of chemotherapy, DCOG ALL-10 protocol day 33, ALL-9 protocol day 42, COALL day 28; TP2, before consolidation, day 79.

^c^Prednisone response on day 8 ≥1.0×10^9^ blasts/L (DCOG ALL-8/10 protocols).

^d^Complete remission (CR) after induction (day 33 in DCOG ALL-9/10, day 42 in ALL-8) is defined on morphological grounds by the presence of <5% leukemic blasts and regenerating hematopoiesis.

^e^Common IKZF1 deletions are defined as full deletion (exons 1-8) and deletion of exons 4-7.

^f^Other *IKZF1* deletions included deletion of exons 1, 1-2, 1-3, 2-3, 2-7, 2-8, 4-8, 5 and 7-8.

### Tyrosine kinase fusion cases show poor treatment response to induction therapy and high minimal residual disease levels

**Figure 2:**
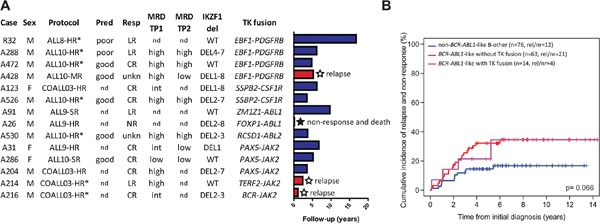
*BCR-ABL1*-like tyrosine kinase fusion cases **A**. Clinical characteristics and follow-up for the tyrosine kinase fusion cases. Barplot representing years from diagnosis to event or censoring. Treatment protocol and arm, prednison window response (Pred), morphological response after induction therapy (Resp), minimal residual disease (MRD), and deletion status of *IKZF1* are shown. MRD monitoring by PCR was performed for research purposes in ALL-9 and COALL03 and for MRD-guided risk stratification in ALL-10. WT indicates no *IKZF1* deletion. For definition of Pred, Resp, and MRD see footnotes Table 3. Response ‘unkn’ indicates patients with low cellularity bone marrow which could not be evaluated. * Indicates patients who received bone marrow transplant, for which HR-treated cases were eligible. **B**. Cumulative incidence of relapse and non-response curves for tyrosine kinase fusion positive *BCR-ABL1*-like cases, fusion-negative *BCR-ABL1*-like cases, and non-*BCR-ABL1*-like B-other cases. Relapse and non-response were considered as events, death as competing event. Cumulative incidence probabilities were estimated using a competing risk model, equality was tested with the Gray test.

Finally, we evaluated clinical response characteristics. Of 12 tyrosine kinase fusion-positive cases with evaluable data, 5 patients did not achieve complete morphological remission at the end induction therapy; 4 of them were late responders, and one was a non-responder resulting in early death (Figure 2A). This non/late response rate at the end of induction therapy was higher in the fusion-positive cases compared with non-*BCR-ABL1*-like B-other (42% vs. 7%, p=0.004) and also higher, albeit not statistically significant, compared with fusion-negative *BCR-ABL1*-like cases (42% vs. 17%, p=0.11; Table 3). Furthermore, fusion-positive cases were characterized by higher levels of minimal residual disease (MRD) compared with non-*BCR-ABL1*-like B-other cases at end of induction therapy (p=0.001) and before consolidation (p=0.002) and also, although less significant, compared with fusion-negative *BCR-ABL1*-like cases (p=0.09, p=0.04, respectively; Table 3). The 8-year cumulative incidence of relapse (CIR) in the *BCR-ABL1*-like group with tyrosine kinase fusions (35% ± 16%) was comparable with the remaining *BCR-ABL1*-like group (35% ± 6%), and worse than the non-*BCR-ABL1*-like B-other group (17% ± 5%; Gray p-value 0.066; Figure 2B). Of the three relapsed fusion-positive cases, one died of relapse and two were alive at end of follow-up (6-12 months). Remarkably, the percentage of males was higher in fusion-positive than fusion-negative *BCR-ABL1*-like cases (79% vs. 44%, p=0.04; Table 3).

## DISCUSSION

We identified ABL/JAK class tyrosine kinase fusion genes in 14/153 (9%) of DCOG/COALL B-other cases and these fusions were restricted to the *BCR-ABL1*-like B-other subgroup. Given that these 153 cases were derived from a population-based selection of BCP-ALL [5] and that these fusions do not co-occur with other driver leukemic fusions (*BCR-ABL1*, rearranged *MLL*, *TCF3-PBX1*, *ETV6-RUNX1*), ABL/JAK class tyrosine kinase fusions cases are estimated to represent approximately 2.5% of pediatric BCP-ALL, making this heterogeneous subgroup similar in size to the *BCR-ABL1*-positive cytogenetic subtype. Although the number of cases evaluated in our study is modest, the frequency of tyrosine kinase fusion cases is similar to those reported in other cohorts: 3% ABL/JAK class fusions in a high-risk pediatric US cohort [6] and 5% ABL/JAK class fusions among Japanese pediatric B-other cases [9].

The most comprehensive detection of novel tyrosine kinase fusion genes requires transcriptome or whole genome sequencing. These costly methods have not been applied to complete cohorts but rather to subsets of cases, for example cases selected based on Philadelphia-like gene expression or absence of sentinel chromosomal abnormalities, mostly in retrospective studies [6, 9]. More targeted approaches to detect recently identified tyrosine kinase fusions include RT-PCR [9], capture-targeted sequencing [17] or targeted locus amplification (TLA) and sequencing [18]. We combined genomic information from copy number, gene expression, targeted RT-PCR, FISH, and TLA to detect tyrosine kinase fusion genes in a representative cohort of 153 BCP-ALL cases without sentinel chromosomal aberrations that were taken from a population-based cohort of 574 cases at initial diagnosis of BCP-ALL. We found tyrosine kinase fusions exclusively in the *BCR-ABL1*-like B-other group, and found them non-overlapping with two chromosomal abnormalities that were described earlier to occur in *BCR-ABL1*-like cases, iAMP21 and dic(9;20) [3]. Moreover, while *CRLF2* high expression cases are present both in *BCR-ABL1*-like and non-*BCR-ABL1*-like B-others, we found that tyrosine kinase fusions and high *CRLF2* expression were mutually exclusive. The current study did not aim at discovering novel TK fusions, therefore there is a small probability that a sample with a novel tyrosine kinase gene is included in the tyrosine kinase fusion negative group.

Our finding that tyrosine kinase fusions are only found in patients with *BCR-ABL1*-like features, and our earlier finding that tyrosine kinase fusion cases are identified by both the St. Jude and Erasmus MC *BCR-ABL1*-like expression signatures [16], means that a pre-screening method based on gene expression, such as the US-developed low density array, is suitable to narrow down the patient population to be screened for tyrosine kinase fusions. An alternative approach screens patients selected by poor early clinical response for tyrosine kinase fusions [10].

We found that ABL/JAK kinase fusion cases were characterized by poor initial response to induction treatment, high MRD levels, and a higher relapse rate compared with non-*BCR-ABL1*-like B-other ALL cases but a comparable relapse rate as *BCR-ABL1*-like ALL without tyrosine kinase fusions. In correspondence, a recent Japanese study showed that the event-free survival of tyrosine kinase-activating fusion cases (albeit including *CRLF2* rearrangements) was unfavorable compared with fusion-negative BCP-ALL [9], Schwab et al. described that *EBF1-PDGFRB*-positive patients were MRD positive at the end of induction [10], and Roberts et al. described MRD positivity and inferior survival in the total group of Philadelphia-like cases [6]. Recent protocols using MRD-based risk-directed therapy suggest that initial poor treatment response in tyrosine kinase fusion-positive cases or the total group of Philadelphia-like cases can be overcome with intensive chemotherapy, leading to durable remission [10, 19]. *Ex vivo* leukemic patients’ cells with JAK and ABL class fusions were shown to be sensitive to tyrosine kinase inhibitors [6–7, 15]. Promising early clinical results suggests that ABL class tyrosine kinase fusion patients respond well to ABL class inhibitors imatinib or dasatinib [6, 10, 20–21]. Together with our data, these reports stress the importance of prospective detection of tyrosine kinase fusions and incorporation of tyrosine kinase inhibitors in ALL treatment protocols to improve outcome.

## MATERIALS AND METHODS

### Patient samples

This study comprised 574 children with newly diagnosed BCP-ALL enrolled in consecutive Dutch Childhood Oncology Group trials (DCOG ALL-8, ALL-9 and ALL-10) [5, 22] and German Cooperative ALL trials (COALL 06-97 and 07-03) [23–24]. These patient cohorts were described and analyzed together previously [3, 5]. Written informed consent was obtained from parents or guardians and institutional review boards approved the use of excess of diagnostic material for research purposes. These studies were conducted in accordance with the Declaration of Helsinki. Mononuclear cells were collected using Lymphoprep sucrose gradient centrifugation (Nycomed Pharma, Oslo, Norway) from bone marrow aspirates and peripheral blood samples obtained prior to treatment. Where needed, mononuclear cells were enriched to >90% leukemic cells by depleting normal cells using anti-CD marker coated magnetic beads (Dynal, Oslo, Norway) as described previously [25]. DNA and total RNA were isolated using TRIzol (Invitrogen Life Technologies, Breda, the Netherlands) and quantified using a Nanodrop ND1000 (Nanodrop, Wilmington, DE). From this cohort, we focused on B-other patients, defined as patients without the sentinel chromosomal abnormalities *BCR-ABL1*, *MLL* rearrangements, *ETV6-RUNX1*, *TCF3-PBX1*, and high hyperdiploidy. Among the B-other cases, *BCR-ABL1*-like cases identified by hierarchical clustering of gene expression data were described previously in our cohort [5]. For 153 out of 204 B-other/*BCR-ABL1*-like cases in the cohort, tyrosine kinase fusion testing was performed (see flow chart in Supplementary Figure 1).

### Tyrosine kinase fusion detection

Detection of tyrosine kinase fusion genes was performed by RT-PCR followed by Sanger sequencing for seven fusion transcripts *EBF1-PDGFRB*, *PAX5-JAK2*, *NUP214-ABL1*, *RANBP2-ABL1*, *ETV6-ABL1*, *RCSD1-ABL1* and *STRN3-JAK2* described by Roberts et al. [7]. In addition, we performed a more extensive RT-PCR panel [6] enabling detection of 30-39 fusion genes (depending on availability of material). For RT-PCR primers see Supplementary Table 2. We used targeted locus amplification for 21 cases to detect fusion genes involving *ABL1*, *PDGFRB*, *CSF1R*, *ABL2*, *TYK2*, and *JAK2* (TLA, Cergentis, Utrecht, the Netherlands) [18, 26]. We used break apart FISH with *PDGFRB*/*CSF1R* and *ABL1* probes (Cytocell) to confirm fusions. The methods applied to each case depended on the type and amount of available patient material and are indicated in Supplementary Table 3 and summarized in Supplementary Figure 1. For the comparison of tyrosine kinase fusion positive versus negative patients, we included 153 samples that were tested at least by the 7-fusion RT-PCR panel. For expression rank analysis (Table 2), the Affymetrix microarray gene expression values for each tyrosine kinase gene were ranked from high to low for the 153 samples. Then the percentile rank of the fusion-positive case among the 153 samples was calculated.

### Genome-wide DNA copy number arrays (array-CGH)

Copy number analysis was performed using Agilent SurePrint G3 Hmn 4×180K arrays (Agilent Technologies, Amstelveen, the Netherlands) co-hybridized with 1 μg patient DNA labeled with ULS-Cy5 and 1 μg reference genomic DNA male pool (G147A, Promega, Leiden, the Netherlands) labeled with ULS-Cy3 (Agilent Genomic DNA ULS Labeling Kit). Copy number microarray data were normalized using median log ratio in the CGHcall [27] version 2.14.0, centralized using CGHnormaliter [28] version 1.8.0, and segmented and called using CGHcall default settings (−1 for loss, 0 for diploid, 1 for gain and 2 for amplification) in R version 2.14.1.

### Multiplex ligation-dependent probe amplification

The SALSA P335 ALL-*IKZF1* (a3) and the SALSA P202 Multiplex Ligation-dependent Probe Amplification (MLPA) assays (MRC-Holland) were used to identify or confirm genomic lesions on the following genes: *IKZF1*, *CDKN2A*, *CDKN2B*, *ETV6*, *PAX5*, *RB1*, *BTG1* and *EBF1* as described previously [5, 29]. In short, 125 ng of genomic DNA was used to generate DNA fragments with incorporated FAM nucleotides according to the manufacturer's protocol. The amplified fragments were quantified using an ABI-3130 genetic analyzer (Applied Biosystems, Carlsbad, CA). Peak intensities were normalized to the manufacturer's control probes and to a synthetic control reference generated from five normal DNA samples in the same MLPA run. A peak ratio lower than 0.75 was considered a deletion, a ratio between 0.75 and 1.3 was considered normal copy number, a ratio higher than 1.3 was considered a gain in copy number.

### Targeted locus amplification

Targeted Locus Amplification (TLA) combined with deep-sequencing was used to detect fusion genes and sequence mutations in regions up to 100 kb around a pre-selected primer pair by crosslinking of physically proximal genomic sequences as described before [26]. Briefly, DNA and protein in 10-15 million viable leukemic blast cells were crosslinked in a 2% formaldehyde solution. Cells were lysed and DNA was digested with NlaIII, followed by ligation, de-crosslinking and DNA purification. DNA molecules were trimmed with NspI and ligated at a concentration of 5 ng/μl to promote intramolecular ligation to DNA fragments of approximately 2 kb. These chimeric fragments were PCR amplified, sonicated and adaptor-ligated for paired-end high-throughput Illumina sequencing. A total of 31 primer sets targeting 19 recurrently affected genes were designed and multiplexed, including the genes involved in the classical cytogenetic subtypes *MLL*, *RUNX1*, *TCF3*, and *IKZF1*, the tyrosine kinase genes *ABL1*, *ABL2*, *PDGFRB*, *CSF1R*, *JAK1*, *JAK2*, *JAK3*, *FLT3*, and *TYK2*, and the cytokine signaling genes *CRLF2*, *EPOR*, *IL7R*, *TSLP*, *SH2B3*, and *IL2RB* [17].

### Fluorescent in-situ hybridization (FISH)

FISH was performed on interface nuclei using break apart probes (Cytocell, Cambridge, UK) for *PDGFRB* and *ABL1*. The FISH probes for *PDGFRB* overlap with the neighboring *CSF1R* locus. At least 100 interphase nuclei were evaluated.

### Reverse transcriptase PCR (RT-PCR)

cDNA was synthesized from 1 μg total or copy RNA using M-MLV reverse transcriptase and combined oligo-dT and pdN6 priming in 20 μl (Promega, Madison, WI). PCR was performed on 2.5 μl cDNA using Taq polymerase, MgCl_2_ and buffer from Applied Biosystems (Bleiswijk, Netherlands). For primer sequences see Supplementary Table 2, for RT-PCR results per sample see Supplementary Table 3.

### Association with clinical outcome

Cumulative incidence of relapse (CIR) was estimated using a competing risks model. We considered relapse as event, and death as competing event. To test for equality of CIRs, Gray's test has been applied. The CIR probability (pCIR) with standard error was reported. Outcome analyses were performed in R 3.0.1, using the packages cmprsk version 2.2-6 [30], mstate version 0.2.6 [31], and survival version 2.37-4 [32].

## SUPPLEMENTARY FIGURES AND TABLES






