# Supplementary material for: Tyrosine kinase fusion genes in pediatric BCR-ABL1-like acute lymphoblastic leukemia
Source: Oncotarget. 2016 Nov 22;8(3):4618–28. doi: 10.18632/oncotarget.13492 (PMC5354859; doi:10.18632/oncotarget.13492)
Supplement: Supplementary file 1 [file oncotarget-08-4618-s001.pdf]

Tyrosine kinase fusion genes in pediatric *BCR-ABL1*-like acute lymphoblastic leukemia

SUPPLEMENTARY DATA

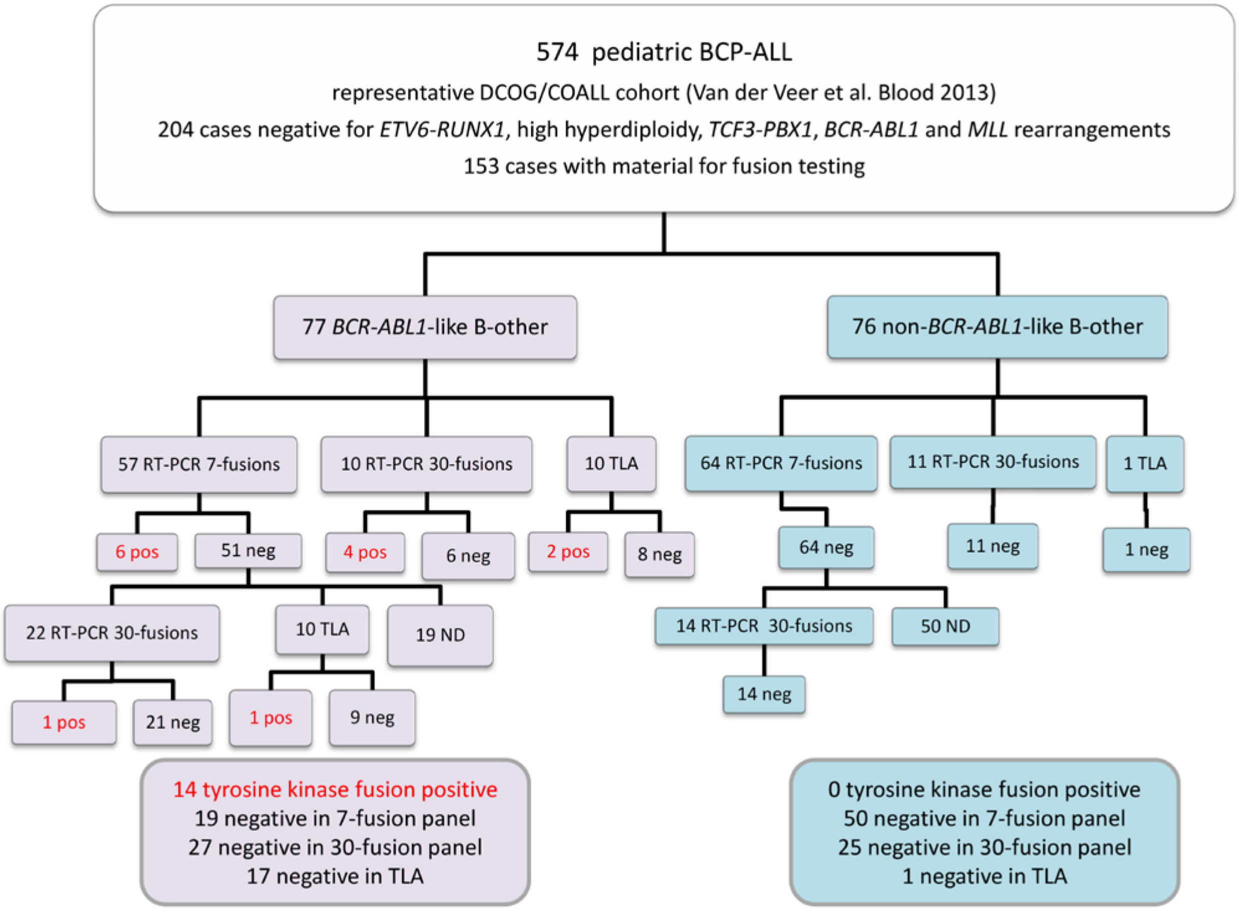

Supplementary Figure 1: Overview of cohort screened for tyrosine kinase fusion genes. Flowchart showing the number of cases tested for tyrosine kinase fusion genes using an RT-PCR panel of 7 fusions [3], an RT-PCR panel of 30 fusions [1], and targeted locus amplification for 6 kinases [4]. Boxes at the bottom summarize the number of positive and negative cases per method. ND, not determined.

A

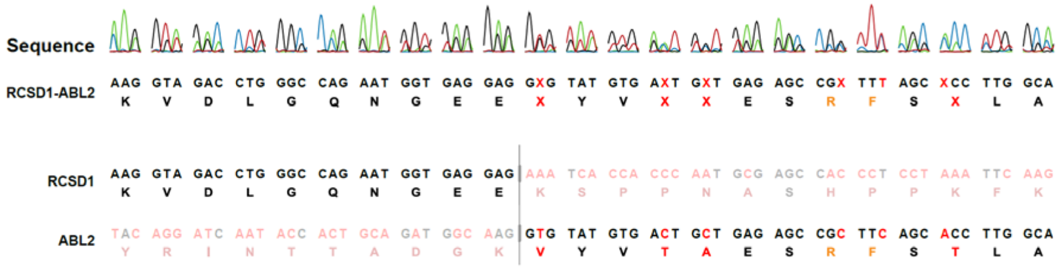

B

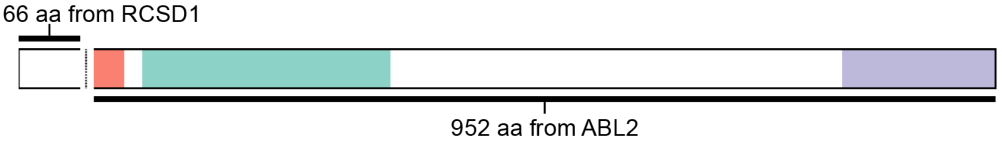

C

AACTAGGCTCCTCCCTGCTCCCTTTTACTGAGGCTCCTTTATGATTTGTCAAGGACACGAACACTATTTTCCAA
GCCTGAGAGAACAGAAAATTGATAATTTTTACAAAGTAGATACTTTATACACTAAAACAAAAGCTTCTTTTTT
TTTT

Supplementary Figure 2: Sanger sequencing and TLA fusion read of *RCSD1-ABL2* in case A530. A. Sequence confirmation of the *RCSD1-ABL2* fusion by Sanger sequencing of RT-PCR product indicates fusion of *RCSD1* exon 3 to *ABL2* exon 5. For primers used in validation RT-PCR see Supplementary Table S2. The fusion encodes an in frame fusion protein. **B.** Schematic representation of the in-frame RCSD1-ABL2 fusion protein drawn in ProteinPaint [5]. **C.** Targeted locus amplification genomic DNA fusion read showing *RCSD1* intron 3 (chr1) (red) and *ABL2* intron 4 (chr1) in green.

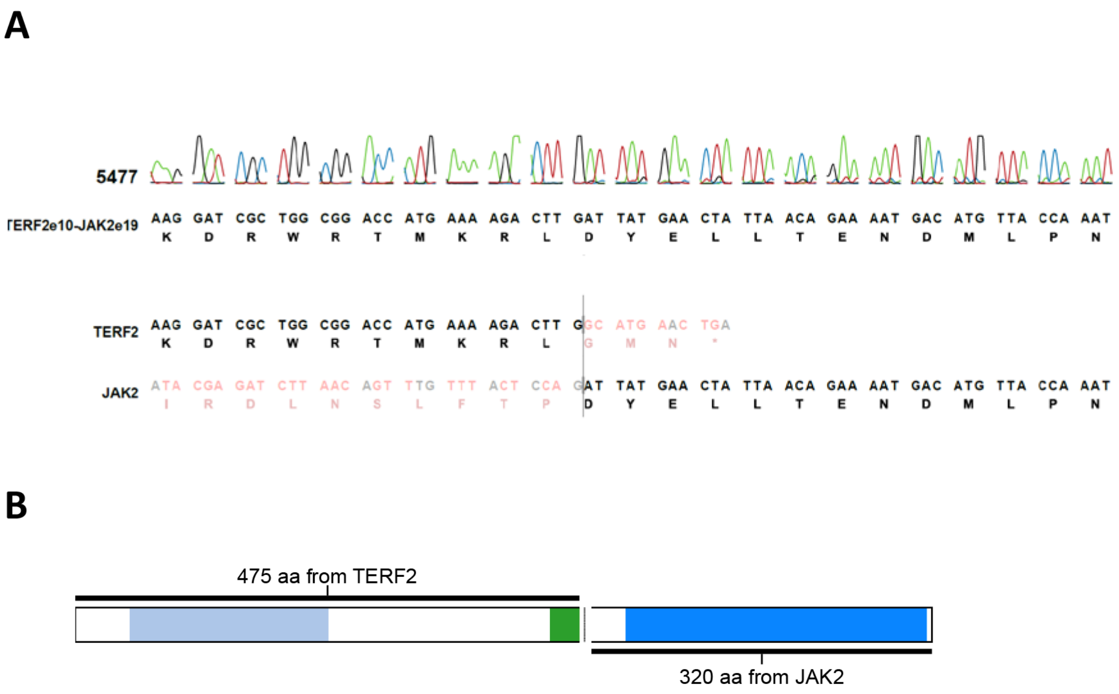

Supplementary Figure 3: Sanger sequencing of *TERF2-JAK2* in case A214. A. Sequence confirmation of the *TERF2-JAK2* fusion by Sanger sequencing of RT-PCR product indicates fusion of *TERF2* exon 10 to *JAK2* exon 19. For primers used in validation RT-PCR see Supplementary Table S2. The fusion encodes an in frame fusion protein. **B.** Schematic representation of the in-frame *TERF2-JAK2* fusion protein drawn in ProteinPaint [5].

(A) *PDGFRB* R32

83% with unbalanced pattern with one normal locus and loss of telomeric probe (Fusion, Red)

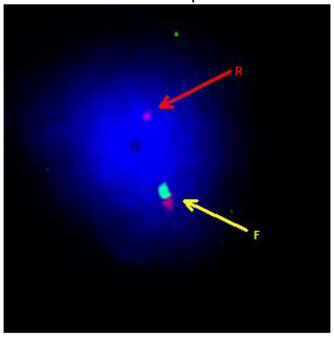

(B) *PDGFRB* A288

32% with balanced translocation and two normal loci (Fusion, Fusion, Red, Green);
56% with balanced pattern and two normal loci and an additional signal for centromeric probe (Fusion, Fusion, Red, Red, Green); also some smaller subclones present

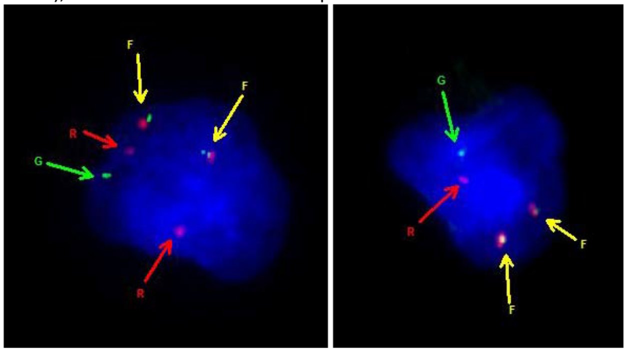

(C) *PDGFRB* A428

22% balanced pattern with one normal locus (Fusion, Red, Green);
64% balanced pattern with one normal locus and one additional signal for telomeric probe (Fusion, Red, Green, Green); some smaller subclones present (Fusion, Fusion, Red, Green; Fusion, Red, Red, Green);

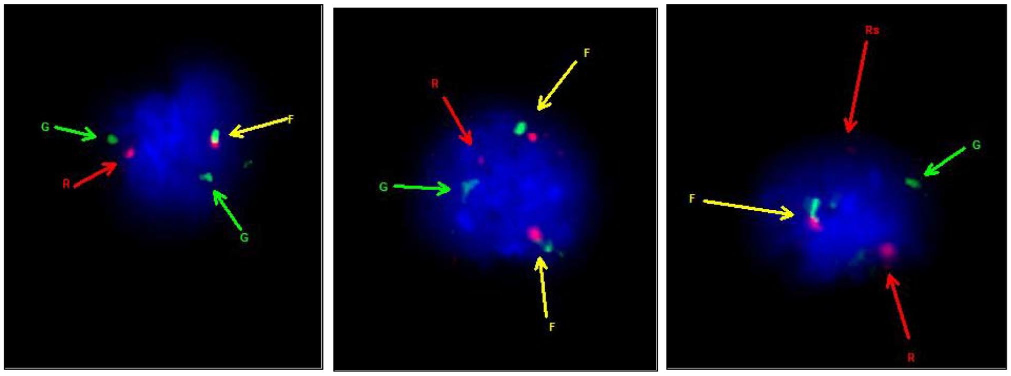

Supplementary Figure 4: FISH with *PDFRGB* break apart probes on *EBF1-PDGFRB* fusion cases. FISH probes were from Cytocell, centromeric probe red, telomeric probe green. **A.** *PDGFRB* R32. 83% with unbalanced pattern with one normal locus and loss of telomeric probe (Fusion, Red). **B.** *PDGFRB* A288. 32% with balanced translocation and two normal loci (Fusion, Fusion, Red, Green); 56% with balanced pattern and two normal loci and an additional signal for centromeric probe (Fusion, Fusion, Red, Red, Green); also some smaller subclones present. **C.** *PDGFRB* A428. 22% balanced pattern with one normal locus (Fusion, Red, Green); 64% balanced pattern with one normal locus and one additional signal for telomeric probe (Fusion, Red, Green, Green); some smaller subclones present (Fusion, Fusion, Red, Green; Fusion, Red, Red, Green);

(A) A288 *EBF1-PDGFRB* balanced translocation t(5;5)

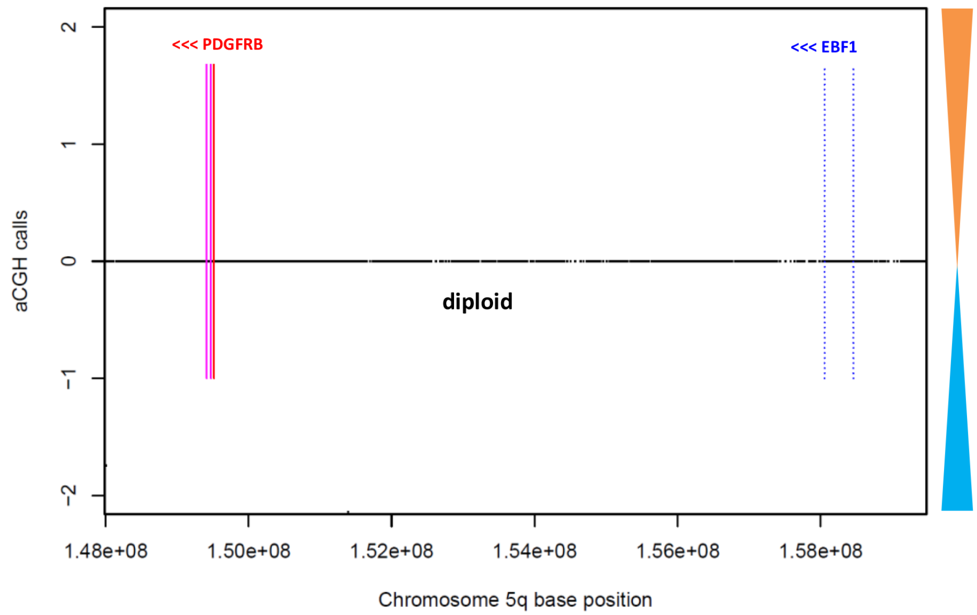

(B) R32 *EBF1-PDGFRB* intrachromosomal deletion on chromosome 5

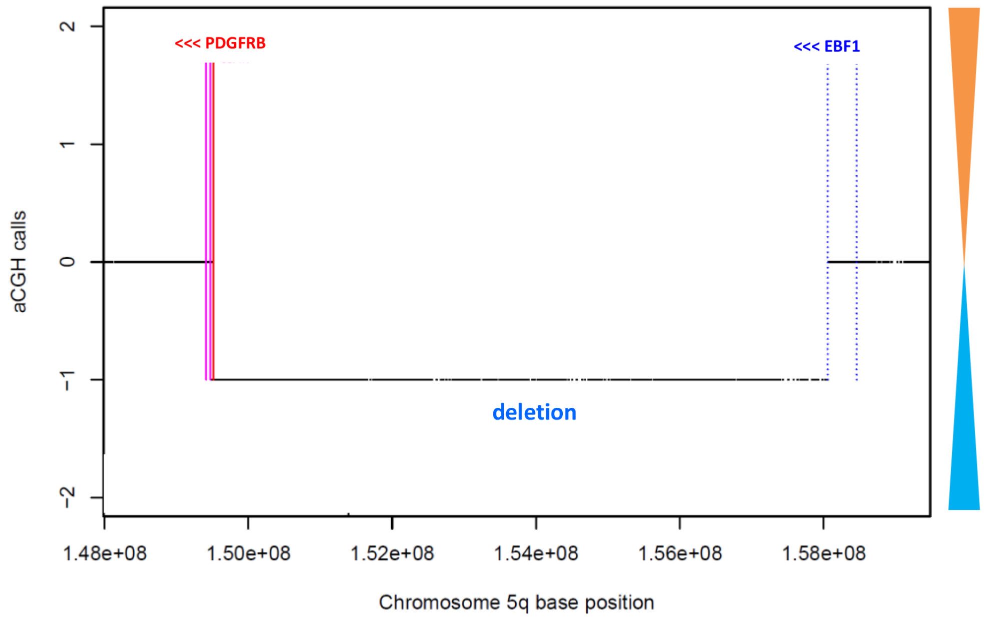

(*Continued*)

(C) A526 *SSBP2-CSF1R* intrachromosomal duplication on chromosome 5

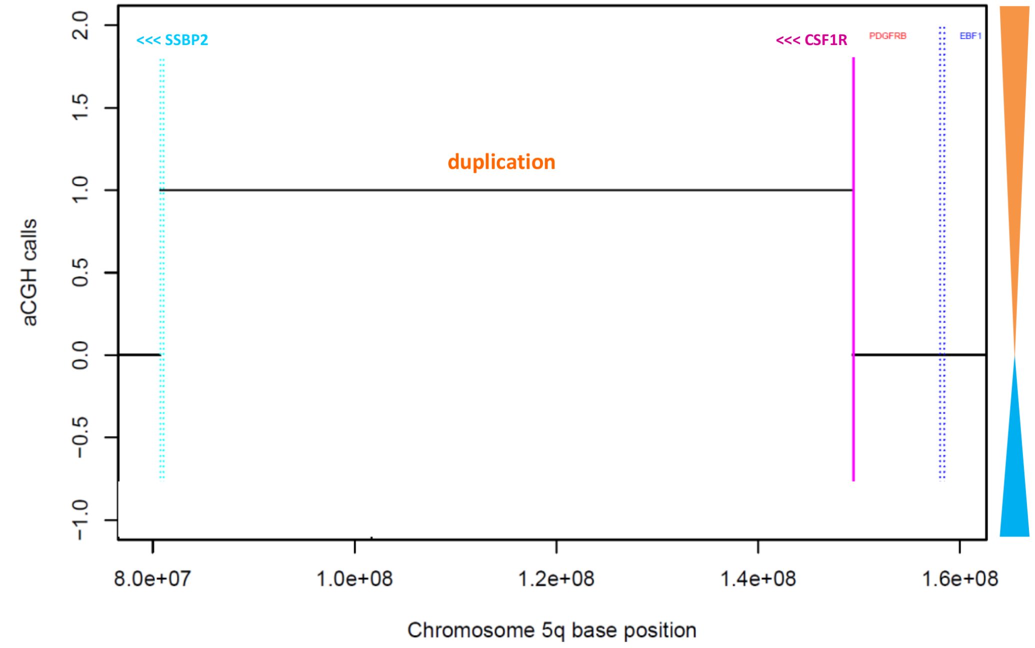

(D) A123 *SSBP2-CSF1R* resulting from chromothripsis of chromosome 5

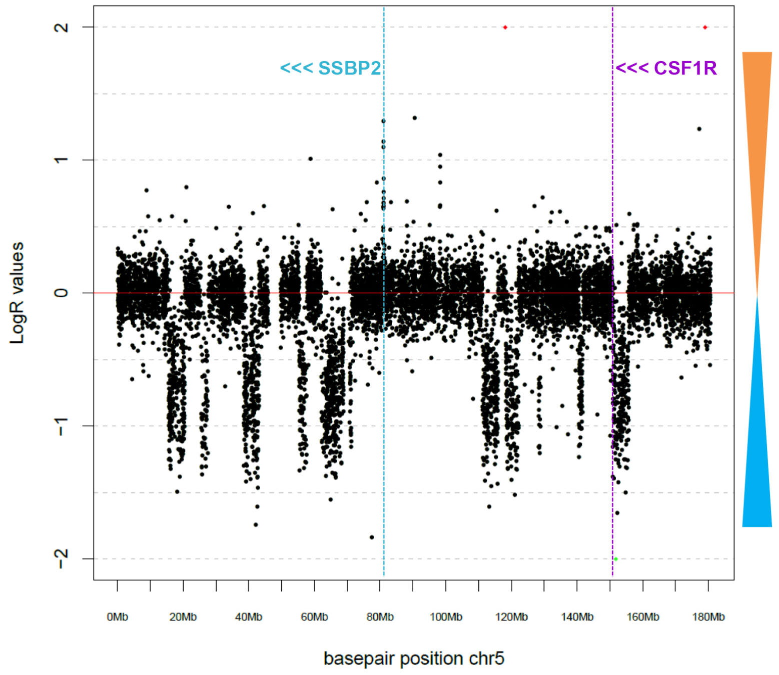

(Continued)

(E) A216 *BCR-JAK2* fusion involving chromosomes 22 and 9

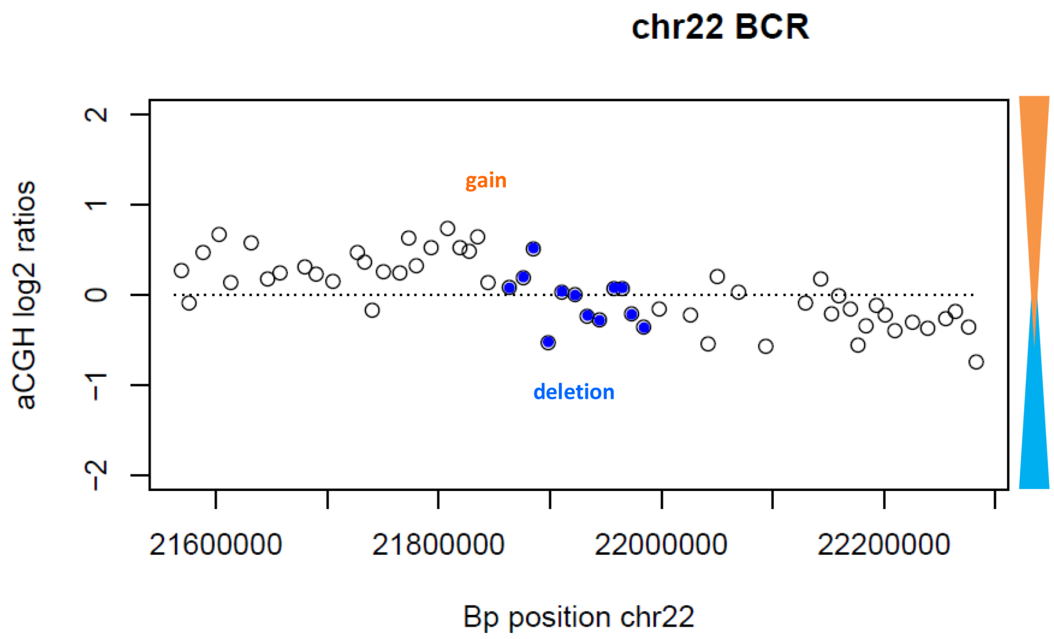

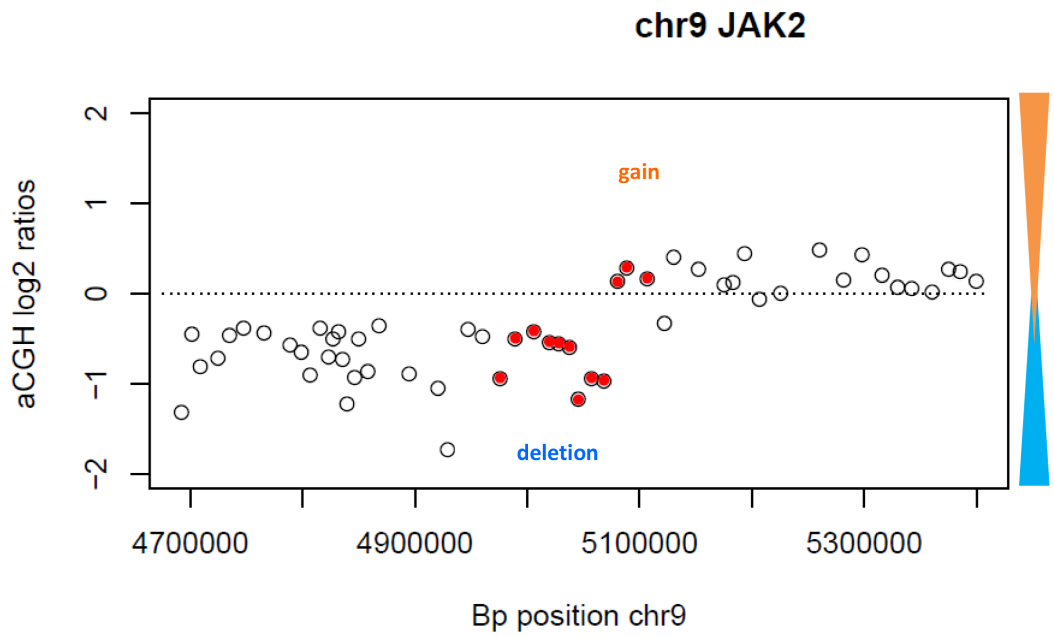

(*Continued*)

(F) A214 *TERF2-JAK2* fusion involving chromosomes 16 and 9

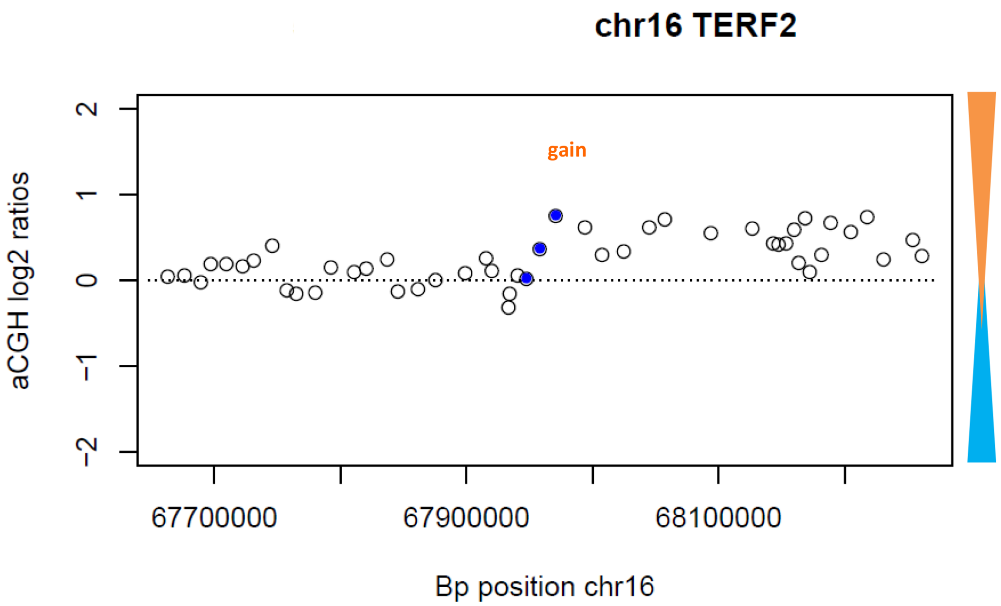

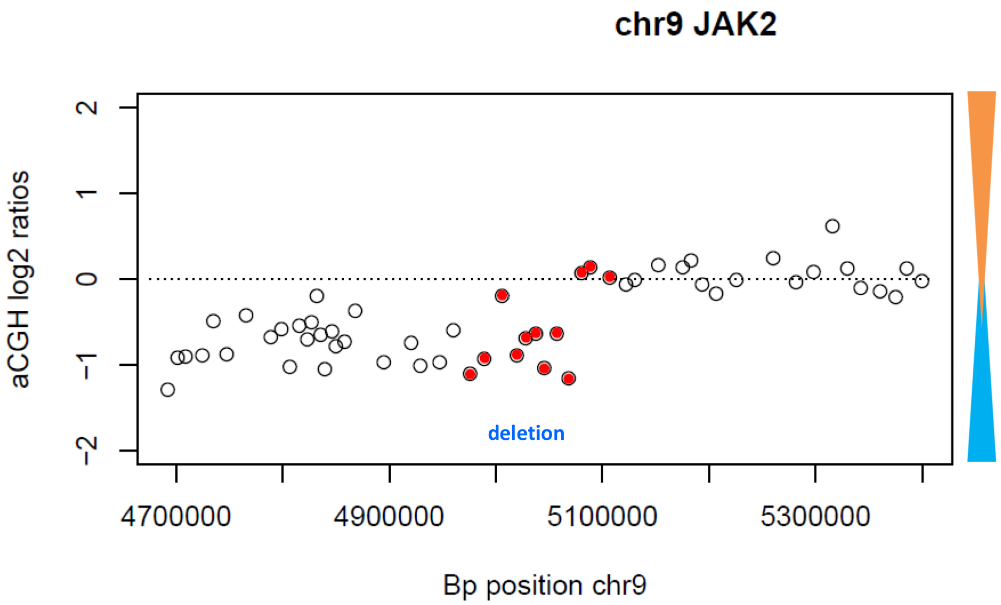

Supplementary Figure 5: Array comparative genomic hybridization 180K Agilent array data for tyrosine kinase fusion cases. The x-axis indicates genomic location on the indicated chromosome, the y-axis shows the called array-CGH data with 0 representing two copies, 1 representing gain and -1 loss **A-C.** or the normalized log2 ratios **D-F.** The colored triangles on the right indicate gain (orange) and loss (blue). (A-D) Rearrangements involving chromosome 5. Beginning and end position of the genes involved in the tyrosine kinase fusions are indicated by colored vertical lines: purple, CSF1R; red, PDGFRB; blue, EBF1; light-blue, SSBP1. The orientation of the genes is indicated with < for minus strand and > plus strand. (E-F) Rearrangements resulting in JAK2 fusions.

Supplementary Table 1: Overview of the numbers of tested and positive cases for the indicated ABL/JAK class tyrosine kinase fusions

Tyrosine kinase fusions	*BCR-ABL1*-like B-other		non-*BCR-ABL1*-like B-other		Total
	tested	positive	tested	positive	tested
EBF1-PDGFRB	77	4	76		153
NUP214-ABL1	77		76		153
RANBP2-ABL1	77		76		153
ETV6-ABL1	77		76		153
RCSD1-ABL1	77		76		153
PAX5-JAK2	77	3	76		153
STRN3-JAK2	77		76		153
TNIP1-PDGFRB	51		23		74
ZEB2-PDGFRB	51		23		74
SSBP2-PDGFRB	51		23		74
SNX2-ABL1	51		23		74
RCSD1-ABL2	51	1	23		74
PAG1-ABL2	51		23		74
ZC3HAV1-ABL2	51		23		74
SSBP2-CSF1R	51	1	23		74
EBF1-CSF1R	51		23		74
ATF7IP-JAK2	51		23		74
EBF1-JAK2	51		23		74
ETV6-JAK2	51		23		74
PPFIBP-JAK2	51		23		74
SSBP2-JAK2	51		23		74
TPR-JAK2	51		23		74
MYH9-IL2RB	51		23		74
MYB-TYK2	51		23		74
TERF2-JAK2	50	1	23		73
ZMIZ1-ABL1	48	1	23		71
PAG1-ABL1	47		23		70
ZC3HAV1-ABL1	47		23		70
NUP214-ABL2	47		23		70
RANBP2-ABL2	47		23		70
ETV6-ABL2	47		21		68
SNX2-ABL2	47		19		66
BCR-JAK2	46	1	23		69
ZMIZ1-ABL2	44		19		63
TNIP1-PDGFRB	43		13		56
ZEB2-PDGFRB	43		13		56
EBF1-CSF1R	43		15		58
BCR-ABL1 (p190)	43		13		56
ETV6-NTRK3	31		22		53
FOXP1-ABL1	19	1	1		20

Supplementary Table 2: Reverse transcription PCR primers used for the detection of tyrosine kinase fusion genes

See Supplementary File 1

Supplementary Table 3: Overview of tyrosine kinase fusion detection on patient cohort

See Supplementary File 2

REFERENCES

1. Roberts KG, Li Y, Payne-Turner D, Harvey RC, Yang YL, Pei D, McCastlain K, Ding L, Lu C, Song G, Ma J, Becksfort J, Rusch M, Chen SC, Easton J, Cheng J, et al. Targetable kinase-activating lesions in Ph-like acute lymphoblastic leukemia. N Engl J Med. 2014; 371:1005-1015.
2. Van der Veer A, Waanders E, Pieters R, Willemse ME, Van Reijmersdal SV, Russel LJ, Harrison CJ, Evans WE, Van der Velden VHJ, Hoogerbrugge PM, Van Leeuwen F, Escherich G, Horstmann MA, Mohammadi Khankahdani L, Rizopoulos D, De Groot-Kruseman HA, et al. Independent prognostic value of BCR-ABL1-like signature and IKZF1 deletion, but not high CRLF2 expression, in children with B-cell precursor ALL. Blood. 2013; 122:2622-2629.
3. Roberts KG, Morin RD, Zhang J, Hirst M, Zhao Y, Su X, Chen SC, Payne-Turner D, Churchman ML, Harvey RC, Chen X, Kasap C, Yan C, Becksfort J, Finney RP, Teachey DT, et al. Genetic alterations activating kinase and cytokine receptor signaling in high-risk acute lymphoblastic leukemia. Cancer Cell. 2012; 22:153-166.
4. Kuiper RP, Van Reijmersdal SV, Simonis M, Yu J, Sonneveld E, Scheijen B, Boer JM, Boeree A, Klous P, Hoogerbrugge P, Yilmaz M, Van Leeuwen FN, Den Boer ML and Splinter E. Targeted locus amplification & next generation sequencing for the detection of recurrent and novel gene fusions for improved treatment decisions in pediatric acute lymphoblastic leukemia. Annual Meeting Abstracts Blood. 2015; 126:696.
5. Zhou X, Edmonson MN, Wilkinson MR, Patel A, Wu G, Liu Y, Li Y, Zhang Z, Rusch MC, Parker M, Becksfort J, Downing JR and Zhang J. Exploring genomic alteration in pediatric cancer using ProteinPaint. Nat Genet. 2015; 48:4-6.
