# Supplementary material for: Tyrosine kinase fusion genes in pediatric BCR-ABL1-like acute lymphoblastic leukemia
Source: Oncotarget. 2016 Nov 22;8(3):4618–28. doi: 10.18632/oncotarget.13492 (PMC5354859; doi:10.18632/oncotarget.13492)
Supplement: Supplementary file 2 [file oncotarget-08-4618-s002.docx]

**Supplemental Table S2. Reverse transcription PCR primers used for the detection of tyrosine kinase fusion genes**

| **Gene fusion** | **Forward Primer ID** | **Forward sequence 5' to 3'** | **Reference Forward** | **Reverse Primer ID** | **Reverse sequence 5' to 3'** | **Reference Reverse** |
| --- | --- | --- | --- | --- | --- | --- |
| ETV6-ABL1 | ETV6_exon5_F4 | ggagaataatcactgcccagcgtcct | Roberts *et al.*, 2014 [1] | ABL1_exon4_R2 | gccaccgtcaggctgtatttcttcc | Roberts *et al.*, 2014 |
| NUP214-ABL1 | NUP_exon20_F5 | cagtggccttggaggaaaacccagt | Roberts *et al.*, 2014 | ABL1_exon4_R2 | gccaccgtcaggctgtatttcttcc | Roberts *et al.*, 2014 |
| RANBP2-ABL1 | RANBP2_exon16_F2 | tggttctttgcgaaatgcagattca | Roberts *et al.*, 2014 | ABL1_exon4_R2 | gccaccgtcaggctgtatttcttcc | Roberts *et al.*, 2014 |
| RCSD1-ABL1 | RCSD1_exon3_F3 | cagccagtaaaccaacccgaaggaa | Roberts *et al.*, 2014 | ABL1_exon4_R2 | gccaccgtcaggctgtatttcttcc | Roberts *et al.*, 2014 |
| SNX2-ABL1 | SNX2_exon3_F1 | cggaaccttctcctgcagtcacacc | Roberts *et al.*, 2014 | ABL1_exon4_R2 | gccaccgtcaggctgtatttcttcc | Roberts *et al.*, 2014 |
| ZMIZ1-ABL1 | ZMIZ1_exon17_forward | gaccggcagatgaaca | Roberts *et al.*, 2014 | ABL1_exon3_reverse | cattccccattgtgattatag | Roberts *et al.*, 2014 |
| PAG1-ABL2 | PAG1_exon7_F1 | ccttcaggagaaggaagggggagagg | Roberts *et al.*, 2014 | ABL2_exon6_R3 | gtcgtggatgggggacacaccatag | Roberts *et al.*, 2014 |
| RCSD1-ABL2 | RCSD1_exon3_F3 | cagccagtaaaccaacccgaaggaa | Roberts *et al.*, 2014 | ABL2_exon6_R3 | gtcgtggatgggggacacaccatag | Roberts *et al.*, 2014 |
| ZC3HAV1-ABL2 | ZC3HAV1_exon11_F2 | tgtcagagatccatcacctacatcca | Roberts *et al.*, 2014 | ABL2_exon6_R3 | gtcgtggatgggggacacaccatag | Roberts *et al.*, 2014 |
| SSBP2-CSF1R | SSBP2_exon12_F2 | tcatgcctagtccagcagattcaacca | Roberts *et al.*, 2014 | CSF1R_exon14_R2 | tggctcatgatcttcagctcggaca | Roberts *et al.*, 2014 |
| MYH9-CSF1R | MYH9_exon1_F2 | gcgggaaggcggcgaggag | Roberts *et al.*, 2014 | CSF1R_exon14_R2 | tggctcatgatcttcagctcggaca | Roberts *et al.*, 2014 |
| MEF2D-CSF1R | MEF2D_e7_F | cctgcgagtcatcacttc | Present study | CSF1R_exon14_R2 | tggctcatgatcttcagctcggaca | Roberts *et al.*, 2014 |
| EBF1-CSF1R | EBF1e11F | ccaccatcgattatggtttccagaggt | Roberts *et al.*, 2014 | CSF1R_exon14_R2 | tggctcatgatcttcagctcggaca | Roberts *et al.*, 2014 |
| ETV6-NTRK3 | ETV6_exon5_F4 | ggagaataatcactgcccagcgtcct | Roberts *et al.*, 2014 | NTRK3_exon15_R1 | atcttgtccttggtcgggctgaggt | Roberts *et al.*, 2014 |
| ATF7IP-JAK2 | ATF7IP_exon12_F2 | aacccatacaaccagcaccgcctct | Roberts *et al.*, 2014 | JAK2_exon20_R4 | tgttgtcatgctgtagggatttcagga | Roberts *et al.*, 2014 |
| BCR-JAK2 | BCR_exon1_F1 | gtgccataagcggcaccggcact | Roberts *et al.*, 2014 | JAK2_exon18_R1 | aggcctgaaatctggttcata | Present study |
| EBF1-JAK2 | EBF _exon14_F2 | cacgagcatgaacggatacggctct | Roberts *et al.*, 2014 | JAK2_exon20_R4 | tgttgtcatgctgtagggatttcagga | Roberts *et al.*, 2014 |
| ETV6-JAK2 | ETV6_exon3_F1 | atggcaaagctctcctgctgctgac | Roberts *et al.*, 2014 | JAK2_exon20_R4 | tgttgtcatgctgtagggatttcagga | Roberts *et al.*, 2014 |
| PAX5-JAK2 | PAX5_exon3_F2 | acaatgacaccgtgcctagcgtcag | Roberts *et al.*, 2014 | JAK2_exon19_R2 | tcaaaggcaccagaaaac | Present study |
| PPFIBP1-JAK2 | PPFIBP1_exon10_F1 | tgcaagatgaaaggagaaggggttga | Roberts *et al.*, 2014 | JAK2_exon20_R4 | tgttgtcatgctgtagggatttcagga | Roberts *et al.*, 2014 |
| SSBP2-JAK2 | SSBP2_exon7_F1 | ggcacttggaggtgtcccaggaagt | Roberts *et al.*, 2014 | JAK2_exon20_R4 | tgttgtcatgctgtagggatttcagga | Roberts *et al.*, 2014 |
| STRN3-JAK2 | STRN3_exon7_F3 | tgaaggagctggagaagcacggagt | Roberts *et al.*, 2014 | JAK2_exon20_R4 | tgttgtcatgctgtagggatttcagga | Roberts *et al.*, 2014 |
| TPR-JAK2 | TPR_exon38_F1 | tggaaatgcctcttccaaagaagttga | Roberts *et al.*, 2014 | JAK2_exon20_R4 | tgttgtcatgctgtagggatttcagga | Roberts *et al.*, 2014 |
| TERF2-JAK2 | TERF2_exon10_F1 | tggggaaggaaactgg | Present study | JAK2_exon20_R4 | tgttgtcatgctgtagggatttcagga | Roberts *et al.*, 2014 |
| EBF1-PDGFRB | EBF1e14 | ccccagcagaccaactat | Present study | PDGFRBe12 | ttccatcggatctcgtaa | Present study |
| TNIP1-PDGFRB | TNIP1_exon13_F2 | aagcactgagcatccaaacc | Roberts *et al.*, 2014 | PDGFRB_exon13_R2 | tttcatcgtggcctgagaatggctc | Roberts *et al.*, 2014 |
| ZEB2-PDGFRB | ZEB2_exon9_F2 | cccaactggagcagctacttttgctga | Roberts *et al.*, 2014 | PDGFRB_exon13_R2 | tttcatcgtggcctgagaatggctc | Roberts *et al.*, 2014 |
| SSBP2-PDGFRB | SSBP2_exon7_F1 | ggcacttggaggtgtcccaggaagt | Roberts *et al.*, 2014 | PDGFRB_exon13_R2 | tttcatcgtggcctgagaatggctc | Roberts *et al.*, 2014 |
| SSBP2-PDGFRB | SSBP2_exon12_F2 | tcatgcctagtccagcagattcaacca | Roberts *et al.*, 2014 | PDGFRB_exon13_R2 | tttcatcgtggcctgagaatggctc | Roberts *et al.*, 2014 |
| MYB-TYK2 | MYB_exon6_F1 | tcaggctccgcctacagctcaactc | Roberts *et al.*, 2014 | TYK2_exon19_R2 | tccgggttcacagtcaagacgtcag | Roberts *et al.*, 2014 |
